# Emergency medicine physicians’ knowledge and perceptions of training, education, and resources in eating disorders

**DOI:** 10.1186/s40337-020-00355-8

**Published:** 2021-01-06

**Authors:** Connie Ma, Diana Gonzales-Pacheco, Jean Cerami, Kathryn E. Coakley

**Affiliations:** Department of Individual, Family, and Community Education, The University of New Mexico, Albuquerque, NM 87131 USA

**Keywords:** Emergency medicine, Feeding and eating disorders, Internship and residency, Emergency service, Hospital, Medical education

## Abstract

**Background:**

Feeding and eating disorders present with a variety of medical complications, some of which may be life-threatening. Emergency Medicine (EM) physicians may interact with patients with eating disorders, however, EM physicians’ knowledge and perceptions of resources for treating patients with eating disorders have not been examined. The purpose of this study was to explore previous training/education, perceptions of available resources, and educational needs in treating eating disorders in practicing EM physicians.

**Methods:**

An investigator-developed survey was used in this cross-sectional pilot study, distributed to EM Residency Program Coordinators in the United States to distribute to EM physicians and residents. The survey assessed EM physicians’ previous training and education in treating and diagnosing eating disorders. The primary outcomes assessed were participants’ previous training/education in eating disorders, knowledge of local resources for patients, and educational needs on a variety of topics related to adult and adolescent eating disorders. Data were described descriptively and SAS 9.4 was used to analyze data.

**Results:**

Of the 162 participants, just 1.9% completed a rotation on eating disorders during residency. Ninety-three percent were unfamiliar with the American Psychiatric Association’s Practice Guideline for the Treatment of Patients with Eating Disorders; 95% were unfamiliar with the publication, “Emergency Department management of patients with eating disorders” by Trent et al. The majority were not aware of resources for patients with eating disorders including community and online support groups, the National Eating Disorders Association, and local treatment programs. At least 50% agreed additional education on 15 of the 19 topics examined would be useful; 85% agreed to wanting education on the assessment of patients with eating disorders in the Emergency Department.

**Conclusions:**

Most EM physicians lack training in eating disorders and knowledge of resources available for patients post-Emergency Department discharge. EM physicians agree additional education on a number of topics would be beneficial, particularly assessment of eating disorders in the Emergency Department, medical complications of eating disorders, and hospital admission criteria for those with eating disorders.

## Plain English summary

This study assessed Emergency Medicine (EM) physicians’ knowledge and perceptions of major eating disorders. Results of the online survey suggest few EM physicians completed education or training on eating disorders during residency or after medical school. The majority of participants were not aware of two relevant patient management guidelines. Participants lacked knowledge of follow-up treatment resources for patients with eating disorders and wanted additional training on a variety of topics, particularly assessment of eating disorders in the Emergency Department, medical complications of eating disorders, and hospital admission criteria for those with eating disorders. Providing EM physicians with additional training and resources for patients with eating disorders could improve the number of patients seeking treatment for an eating disorder post-Emergency Department discharge.

## Background

Eating disorders, specifically anorexia nervosa, have one of the highest mortality rates of all mental illnesses [1]. There are many types of feeding and eating disorders (FEDs) defined by the DSM-5 including anorexia nervosa, bulimia nervosa, binge eating disorder, and other specified feeding and eating disorders (OSFED), each manifesting with a variety of signs and symptoms [2]. The crude mortality rates of major eating disorders are high, ranging from 5.1 deaths per 1000 person-years in anorexia nervosa to 1.7 deaths per 1000 person-years in bulimia nervosa [3]. Eating disorder complications range in severity and include physical and mental health impacts. Medical complications of anorexia nervosa are largely related to starvation and may include amenorrhea, cardiac abnormalities, and osteoporosis [4]. Medical complications of bulimia nervosa are largely related to purging and include electrolyte disturbances resulting in cardiac abnormalities, gastroesophageal reflux and gastrointestinal issues, and even esophageal or gastric rupture [5]. Binge eating disorder may result in weight gain leading to obesity, gastrointestinal issues, and disruptions in endocrine function [5]. All FEDs are also associated with co-morbid mental health disorders including depression, anxiety, obsessive-compulsive disorder, and substance use disorders [6].

Symptoms of eating disorders can be physically and biochemically apparent but may not be linked to disordered eating when patients present to physicians. Individuals with eating disorders may choose not to disclose the eating disorder or do not recognize severity of disordered behaviors [7]. Diagnosis may also be missed due to providers’ lack of education or training regarding eating disorders [7]. A national survey of eating disorder training across five medical specialty residency programs (internal medicine, pediatrics, family medicine, psychiatry, and child and adolescent psychiatry) found that the majority of these programs did not provide any scheduled or elective rotations for eating disorders [8]. Physicians also report low comfort levels when managing patients with eating disorders due to lack of undergraduate and postgraduate training [9].

The Emergency Medicine (EM) specialty represents an opportunity for early identification of individuals with eating disorders. EM physicians may be the first or only provider a patient interacts with. Adolescent and young adult patients with eating disorders with previous visits to the Emergency Department are 1.6 times more likely to visit the Emergency Department than those without eating disorders [10]. In a separate study, more than half of children and adolescents with mental health problems who presented at the Emergency Department had not previously received mental health outpatient services [11]. Emergency Medicine physicians could have a critical role in identifying patients with eating disorders.

The purpose of this study was to explore previous training/education and knowledge of available resources in treating adult and adolescent patients with eating disorders in a sample of practicing EM physicians in the United States. The focus of the investigation was on major eating disorders (anorexia nervosa, bulimia nervosa, binge eating disorder, OSFED), not all FEDs. In addition, future educational needs on topics related to eating disorders were assessed.

## Methods

### Study design and setting

An investigator-developed survey was used to collect data in this cross-sectional pilot study. The survey included basic demographic questions and assessed EM physicians’ previous training, education, knowledge of treatment-related resources, and education and training needs in treating and diagnosing major eating disorders in adults and adolescents. Survey questions were developed using current literature and the final survey was reviewed by the Chair of a University Psychology Department and the National Eating Disorder Association (NEDA). The survey was administered through the online survey platform, Opinio.

### Selection and description of participants

Participants were recruited by email using a snowball sampling approach. The principal investigator first contacted all EM Residency Program Coordinators listed on the American College of Emergency Physicians’ website. This email included a request to forward information about the study and a link to participate to EM residents and physicians in their department and professional networks. Interested participants clicked a link to the survey in the recruitment email and were directed to an informed consent. After consent, participants were screened for eligibility to participate. Inclusion criteria for participation were 1) practicing physician or medical resident and 2) currently working (full-time, part-time, or PRN) in an Emergency Department. Participants were excluded if they did not meet both inclusion criteria. This study received approval from the University of New Mexico Institutional Review Board.

### Measures

The survey was available in Opinio from April to June 2018. Two reminders to participate were sent to EM Program Coordinators to distribute to their faculty and networks at the midpoint and two weeks prior to survey closure. After completion of the survey, participants had the option of emailing the study team to be eligible to receive a $25 incentive. Five participants were randomly chosen at the end of the study to receive a $25 gift card. The data were stored on Opinio.

### Outcomes

To assess prior education and training, participants were asked if they received training on eating disorders in medical school and if they completed a scheduled or elective rotation on eating disorders during residency. Participants could also indicate other education/training they had received on eating disorders and if it was mandatory or elective. Participants were asked if they were familiar with the American Psychiatric Association’s Practice Guideline for the Treatment of Patients with Eating Disorders [12] or Trent et al.’s 2013 publication, “Emergency Department management of patients with eating disorders” in the American Journal of Emergency Medicine [2]. These practice guidelines discuss developing and implementing a treatment plan to include psychiatric management for patients with eating disorders.

To assess knowledge of resources, participants were asked to indicate agreement via Likert scale to being knowledgeable about a variety of resources, treatment options, and eating disorder organizations in their location for patients with suspected or diagnosed eating disorders after an Emergency Department visit. To assess education and training needs, participants were asked to indicate agreement via Likert scale to the usefulness of additional education and training on a variety of eating disorder-related topics.

### Statistical analysis

As this was a pilot study, a power analysis was not conducted. Participants who completed at least 50% of the survey, including at least one of the primary research questions, were eligible for inclusion in the final sample. Data were extracted from Opinio for statistical analyses. SAS 9.4 was used to analyze data. For continuous variables, normality was assessed using the Shapiro-Wilk statistic. For normally distributed linear data, means and standard deviations were reported; for nonparametric data, medians and interquartile range (IQR) were reported. For categorical data, frequencies and percentages were reported. For Likert scale questions, strongly disagree and disagree were combined to “Disagree” and strongly agree and agree were combined to “Agree”. A *p*-value of <0.05 was considered statistically significant.

## Results

### Characteristics of study subjects

**Fig. 1:**
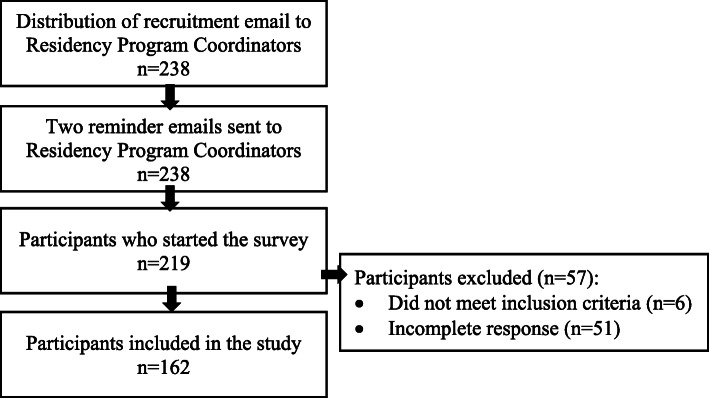
Summary of participant recruitment

Two-hundred thirty-eight residency programs were contacted by email to distribute the survey to their EM faculty and networks. Figure 1 includes a summary of participant recruitment. Over the four-week period the survey was available, 219 participants started the survey. Six did not meet inclusion criteria and 51 did not complete the survey, leaving 162 (74%) participants in the final sample (Table 1). Of the 162 participants, 150 completed all survey questions.

**Table 1:** Demographic characteristics of participants (*n* = 162)

Characteristic	Median (IQR)	n	%
Age (years)	31 (29–35)		
Gender			
Male		78	48.2%
Female		84	51.8%
Race/Ethnicity			
African American		4	2.5%
Asian/Pacific Islander		20	12.3%
Caucasian		118	72.8%
Hispanic		6	3.7%
Native American		0	0%
Other		5	3.1%
> 1 Race/Ethnicity		9	5.6%
Geographic region of practice			
Northeast		31	19.1%
Midwest		61	37.7%
South		30	18.5%
West		38	23.5%
Not reported		2	1.2%
Full-time practice (years)	3 (1–5)		
Full-time practice in ED (years)	3 (1–5)		
Experience in the ED			
< 5 years		116	71.6%
≥ 5 years		41	25.3%
Not reported		5	3.1%

*Abbreviations*: *IQR* interquartile range, *ED* Emergency Department

Of the 162 participants, median age was 31 years (range 25–65 years). The majority were female (*n* = 84; 51.8%), Caucasian (*n* = 118; 72.8%), and had practiced for less than 5 years in the Emergency Department (*n* = 116; 71.6%). In the sample of participants, 23 states were represented. The majority of participants reported practicing primarily in California (*n* = 21), Ohio (*n* = 19), Missouri (*n* = 17), New York (*n* = 15), or Arizona (*n* = 13). Based on Census Bureau defined regions, the majority of participants practiced in the Midwest (*n* = 61; 37.7%).

The majority of participants reported receiving training on eating disorders in medical school (*n* = 138; 85.7%). Of those who did receive training on eating disorders in medical school, 68 (49.3%) reported training was inadequate. Only three participants (1.9%) reported completing a scheduled or elective rotation on eating disorders during residency. The majority (*n* = 152; 93.8%) reported they did not complete a scheduled or elective rotation on eating disorders during residency because it was not offered.

### Main results

Most respondents were not familiar with the American Psychiatric Association’s Practice Guideline for the Treatment of Patients with Eating Disorders (*n* = 151; 93.2%) or the Trent et al. publication, “Emergency Department management of patients with eating disorders” (*n* = 154; 95.1%).

The majority of respondents were not knowledgeable about resources in their location for patients with suspected or diagnosed eating disorders after an Emergency Department visit. In particular, participants disagreed to being knowledgeable about the Alliance for Eating Disorder Awareness (74.2%), community support groups (73.2%), or online support groups (72%) (Table 2). Most respondents (79%) agreed to being knowledgeable about following up with an appropriate primary care physician after a patient with a suspected or diagnosed eating disorder leaves the Emergency Department.

**Table 2:** Physicians’ agreement to knowledge of resources for patients with eating disorders after their Emergency Department visit

Resources	Disagree		Neutral		Agree		Total
	n	%	n	%	n	%	
Alliance for Eating Disorder Awareness	115	74.2	23	14.8	17	11.0	155
Community support groups	115	73.2	21	13.4	21	13.6	157
Online support groups	113	72.0	26	16.6	18	11.5	157
The National Eating Disorders Association	111	70.7	27	17.2	19	12.1	157
Outpatient treatment programs (partial hospitalization program, intensive outpatient program)	107	68.2	28	17.8	22	14.0	157
Residential treatment programs	101	64.3	30	19.1	26	16.6	157
Self-help materials	96	61.1	40	25.5	21	13.4	157
Patient education materials/discharge instructions	41	26.1	37	23.6	79	50.3	157
Outpatient follow up (psychiatrist, psychologist, licensed therapist, dietitian, other)	37	23.6	27	17.2	93	59.2	157
Follow up with primary care physician	12	7.6	21	13.4	124	79.0	157

*Abbreviation*: *ED* Emergency Department

The majority of respondents (> 50%) agreed additional education on 15 of the 19 topics included would be useful. The top three educational needs were: assessment of patients with eating disorders in the Emergency Department (85% agreement), medical complications (83.7% agreement), and suggested criteria for hospital admission (82.8% agreement). The fewest agreed additional education on food addiction (43.2% agreement) would be useful (Table 3).

**Table 3:** Physicians’ agreement to usefulness of additional education and training on eating disorder-related topics

Topics	Disagree		Neutral		Agree		Total
	n	%	n	%	n	%	
Assessment of patients with eating disorders in the ED	3	2.0	19	12.9	125	85.0	147
Medical complications	3	2.0	21	14.3	123	83.7	147
Suggested criteria for hospital admission	3	2.1	22	15.2	120	82.8	145
Resources for patients with eating disorders	4	2.7	29	19.6	115	77.7	148
Treatment options after ED discharge	5	3.4	30	20.3	113	76.4	148
Adult eating disorders	4	2.7	33	22.3	111	75.0	148
Pediatric eating disorders	7	4.7	31	20.9	110	74.3	148
Diagnosis of eating disorders	7	4.8	31	21.1	109	74.1	147
Diabulimia	9	6.1	50	34.0	88	59.9	147
SCOFF questionnaire for screening	18	12.3	43	29.5	85	58.2	146
Bulimia nervosa	12	8.2	52	35.4	83	56.5	147
Severe and enduring anorexia nervosa	18	12.4	47	32.4	80	55.2	145
Anorexia nervosa restrictive type	18	12.2	50	33.8	80	54.1	148
Anorexia nervosa binge-purge subtype	18	12.3	50	34.2	78	53.4	146
Binge eating disorder	18	12.3	52	35.6	76	52.1	146
Orthorexia	17	11.6	61	41.5	69	46.9	147
Avoidant/ restrictive food intake disorder	16	10.9	63	42.9	68	46.3	147
Other specified feeding or eating disorder	19	13.0	61	41.8	66	45.2	146
Food addiction	24	16.2	60	40.5	64	43.2	148

*Abbreviations*: *ED* Emergency Department, *SCOFF Questionnaire* Sick, Control, One, Fat, Food

## Discussion

This is the first study examining knowledge and perceptions of eating disorders, and educational needs, in EM physicians in the United States. Results suggest eating disorder training in medical school was provided but not during residency and that EM clinicians need more information and education on available resources for patients with diagnosed or suspected eating disorders.

Overall, most EM physicians were unaware of two clinical practice guidelines: the Practice Guideline for the Treatment of Patients With Eating Disorders published in 2006 by the American Psychiatric Association (APA) [12] and the peer-reviewed article, “Emergency Department management of patients with eating disorders” published in 2013 by Trent et al. [2]. The APA Guidelines are a gold-standard resource for all types of physicians; however, Trent et al. Guidelines are specific to EM providers. Comprehensive treatment and prospective management of patients with eating disorders does not occur in the Emergency Department, however, EM clinicians can draw from guidelines to develop hospital admission criteria and/or provide follow-up referrals. Knowledge of guidelines and follow-up treatment options are critical as recent studies suggest 15.9% of adult participants screened positive for an eating disorder in the Emergency Department [13] and 16% of adolescent and young adults screened positive for an eating disorder in an Emergency Department [14]. One study of Australian EM, general, pediatric, and psychiatric clinicians reported previous knowledge of the 2014 Royal Australian and New Zealand College of Psychiatrists clinical practice guidelines for the treatment of eating disorders was related to increased confidence in treating eating disorders [15]. The APA and the Trent et al. guidelines are open access. Guidelines may also be incorporated into medical school, residency, and fellowship program curriculum.

In the present study, a very high percentage of physicians did not know about resources in their community for patients with eating disorders post-discharge from the Emergency Department including treatment programs, support groups, and non-profit organizations. Awareness of treatment options post-Emergency Department discharge is essential so that physicians may provide recommendations for follow-up specialty care or referrals to reduce comorbidities of eating disorders. Providing follow-up recommendations to patients and families requires little extra time on the part of the EM clinician and patients may be more likely to seek treatment. If barriers were to exist in the regional area for referrals to eating disorder treatment centers, such as poor accessibility to specialist services, then Emergency Department clinicians including physicians, nurses, social workers, and case managers should be aware of affordable and easily assessable resources on the internet or in nearby communities or states [15]. NEDA and the Alliance for Eating Disorder Awareness are two non-profit organizations focusing on advocacy, evidenced-based research, and providing assistance finding treatment for individuals and families who are affected by eating disorders.

Most respondents wanted more education on assessment of eating disorders in the Emergency Department, medical complications of eating disorders, and hospital admission criteria for those with eating disorders. Interestingly, one study found that eight out of nine physicians who specialized in mental health, EM, and other specialties, were competent in assessment of eating disorders [15]. A separate study by Linville et al. also examined educational needs of clinicians (obstetricians and gynecologists, family practice physicians and nurse practitioners, general physicians and nurse practitioners, and pediatricians and pediatric nurse practitioners) related to eating disorders [16]. Respondents indicated that it was difficult for them to successfully screen and treat patients when they were uncertain about the course of treatment or did not have sufficient previous knowledge and training on eating disorders. Educational needs identified in this study and the study by Linville et al. are important to consider implementing in medical training programs and curriculum.

Linville et al. found clinicians wanted a brief screening tool to help identify and diagnose eating disorders [16]. Respondents in this study were also interested in a brief screening tool, specifically the SCOFF questionnaire, developed by Morgan et al. in 1999 [17]. The SCOFF questionnaire has five questions and is a quick tool utilized by many health professions to screen for eating disorders [18–20]. It is also recommended in the Trent et al. guidelines for EM clinicians. According to a recent systematic review and meta-analysis, however, the SCOFF questionnaire warrants more studies before being used to screen for eating disorders in primary care and community-based settings [18]. The SCOFF questionnaire is currently available for free on NEDA’s website.

The present study has limitations. Approximately 25% of participants who started the survey did not complete it. The number of individuals who received the recruitment email to participate was also not assessed since the study used a snowball sampling approach. Due to the small sample size, results may not be generalizable to the general population of practicing EM physicians. Selection bias could have also occurred as those who decided to complete the survey may have stronger feelings about eating disorders than those who did not participate or complete the survey. Most respondents (71.6%) had also practiced less than 5 years in the Emergency Department, potentially introducing bias related to lack of awareness of resources and treatment options for patients with eating disorders. Although data indicate EM physicians lack training and education on eating disorders, results may not reflect the entire EM specialty as not all 50 states of the US were represented.

## Conclusions

In summary, these findings add to the limited literature about EM physicians and eating disorders. Future research may include a larger sample of EM physicians and include the provision of education to determine if additional training on eating disorders improves recognition of patients with signs and symptoms and referral to appropriate follow-up treatment. Providing EM physicians with comprehensive education, training, and awareness of resources regarding eating disorders could result in potentially identifying more patients with eating disorders and providing important follow-up resources.

## Supplementary Information

**Additional file 1.**

## Data Availability

The dataset analyzed during the current study is available from the corresponding author on reasonable request.

## Abbreviations

FED: Feeding and eating disorder
OSFED: Other specified feeding and eating disorders
APA: American Psychiatric Association
EM: Emergency Medicine
IQR: Interquartile Range
NEDA: The National Eating Disorders Association
SCOFF: Sick, Control, One, Fat, Food

## References

[1] Chesney E, Goodwin GM, Fazel S (2014). Risks of all-cause and suicide mortality in mental disorders: a meta-review. World Psychiatry.

[2] Trent SA, Moreira ME, Colwell CB, Mehler PS (2013). ED management of patients with eating disorders. Am J Emerg Med.

[3] Smink FR, van Hoeken D, Hoek HW (2013). Epidemiology, course, and outcome of eating disorders. Curr Opin Psychiatry.

[4] Rome ES, Ammerman S (2003). Medical complications of eating disorders: an update. J Adolesc Health.

[5] Voderholzer U, Haas V, Correll CU, Körner T (2020). Medical management of eating disorders: an update. Curr Opin Psychiatry.

[6] O'Brien KM, Vincent NK (2003). Psychiatric comorbidity in anorexia and bulimia nervosa: nature, prevalence, and causal relationships. Clin Psychol Rev.

[7] Anderson K, Accurso EC, Kinasz KR, Le Grange D (2017). Residents’ and fellows’ knowledge and attitudes about eating disorders at an Academic Medical Center. Acad Psychiatry.

[8] Mahr F, Farahmand P, Bixler EO, Domen RE, Moser EM, Nadeem T (2015). A national survey of eating disorder training. Int J Eat Disord.

[9] Boulé CJ, McSherry JA (2002). Patients with eating disorders. How well are family physicians managing them?. Can Fam Physician.

[10] Dooley-Hash S, Lipson SK, Walton MA, Cunningham RM (2013). Increased emergency department use by adolescents and young adults with eating disorders. Int J Eat Disord.

[11] Gill PJ, Saunders N, Gandhi S, Gonzalez A, Kurdyak P, Vigod S (2017). Emergency Department as a first contact for mental health problems in children and youth. J Am Acad Child Adolesc Psychiatry.

[12] Yager J, Devlin MJ, Halmi KA, Herzog DB, Mitchell JE, Powers P, et al. Practice guideline for the treatment of patients with eating disorders. 3rd edn Philadelphia: American Psychiatric Association (APA); 2006.

[13] Dooley-Hash S, Adams M, Walton MA, Blow FC, Cunningham RM (2019). The prevalence and correlates of eating disorders in adult emergency department patients. Int J Eat Disord.

[14] Dooley-Hash S, Banker JD, Walton MA, Ginsburg Y, Cunningham RM (2012). The prevalence and correlates of eating disorders among emergency department patients aged 14-20 years. Int J Eat Disord.

[15] Lakeman R, McIntosh C (2018). Perceived confidence, competence and training in evidence-based treatments for eating disorders: a survey of clinicians in an Australian regional health service. Australas Psychiatry.

[16] Linville D, Benton A, O'Neil M, Sturm K (2010). Medical providers’ screening, training and intervention practices for eating disorders. Eat Disord.

[17] Morgan JF, Reid F, Lacey JH (2000). The SCOFF questionnaire: a new screening tool for eating disorders. West J Med.

[18] Kutz AM, Marsh AG, Gunderson CG, Maguen S, Masheb RM (2020). Eating disorder screening: a systematic review and meta-analysis of diagnostic test characteristics of the SCOFF. J Gen Intern Med.

[19] Solmi F, Hatch SL, Hotopf M, Treasure J, Micali N (2015). Validation of the SCOFF questionnaire for eating disorders in a multiethnic general population sample. Int J Eat Disord.

[20] Maguen S, Hebenstreit C, Li Y, Dinh JV, Donalson R, Dalton S (2018). Screen for disordered eating: improving the accuracy of eating disorder screening in primary care. Gen Hosp Psychiatry.

