# Supplementary material for: Emergency medicine physicians’ knowledge and perceptions of training, education, and resources in eating disorders
Source: J Eat Disord. 2021 Jan 6;9:4. doi: 10.1186/s40337-020-00355-8 (PMC7789763; doi:10.1186/s40337-020-00355-8)
Supplement: Supplementary file 1 — Additional file 1. [file 40337_2020_355_MOESM1_ESM.docx]

Physician Knowledge of Eating Disorders in Patients Presenting to the Emergency Department

***Demographic Questions:***

1. Age: ______________
2. Sex:
   1. Male
   2. Female
   3. Other _______________________
3. Race/Ethnicity (you may select more than one):
   1. African American
   2. Asian/Pacific Islander
   3. Caucasian
   4. Hispanic
   5. Native American
   6. Other __________________________
4. Medical School attended: __________________
5. Year of graduation from Medical School: ________________________
6. Residency Training Program completed or in progress (if applicable): ________________________
7. Residency Training Program Specialty (if applicable): ________________________
8. Year of completion of Residency Training Program (actual or anticipated): ________________
9. Current State(s) in which you practice regularly: ________________________
10. Current Institution(s) of Practice: ________________________
11. Total number of years of full-time equivalent practice: ________________________
12. Total number of years of full-time equivalent practice in the ED: ________________________

***ED Training/Education:***

1. I received training on eating disorders in medical school.
   1. Yes—training was adequate.
   2. Yes—training was inadequate.
   3. No—by choice.
   4. No—not offered.
2. I completed a scheduled rotation on eating disorders during my residency.
   1. Yes—training was adequate.
   2. Yes—training was inadequate.
   3. No—by choice.
   4. No—not offered.
   5. N/A
3. I completed an elective rotation on eating disordering during my residency.
   1. Yes—training was adequate.
   2. Yes—training was inadequate.
   3. No—by choice.
   4. No—not offered.
   5. N/A
4. Length of rotation (weeks): ___________________________
5. The rotation prepared me to treat patients in the ED with suspected or diagnosed eating disorders.
   1. Strongly disagree
   2. Disagree
   3. Neutral
   4. Agree
   5. Strongly agree
   6. N/A
6. Other education/training (including Continuing Medical Education) completed on eating disorders in pediatric or adult patients: ________________________________________________________
7. If Question 6 is applicable, was this training mandatory or optional?
   1. Mandatory
   2. Optional
   3. N/A
   4. Other ____________________
8. Prior to this survey, were you familiar with the American Psychiatric Association’s Practice Guideline for the Treatment of Patients with Eating Disorders? (<http://psychiatryonline.org/pb/assets/raw/sitewide/practice_guidelines/guidelines/eatingdisorders.pdf>).
   1. Yes
   2. No
9. Prior to this survey, were you familiar with Trent et al.’s 2013 publication, “ED management of patients with eating disorders” in the American Journal of Emergency Medicine? (<https://www.ncbi.nlm.nih.gov/pubmed/23623238>).
   1. Yes
   2. No

I am knowledgeable about the following resources in my location for patients with suspected and diagnosed eating disorders after their ED visit:

1. Follow-up with an appropriate primary care physician

Strongly disagree Disagree Neutral Agree Strongly agree

1. Follow-up with appropriate members of an outpatient treatment team (psychiatrist, psychologist, licensed therapist, dietitian, other)

Strongly disagree Disagree Neutral Agree Strongly agree

1. Residential treatment programs for eating disorders

Strongly disagree Disagree Neutral Agree Strongly agree

1. Outpatient treatment programs for eating disorders (partial hospitalization program, intensive outpatient program)

Strongly disagree Disagree Neutral Agree Strongly agree

1. Patient education materials/discharge instructions

Strongly disagree Disagree Neutral Agree Strongly agree

1. Community support groups for eating disorders

Strongly disagree Disagree Neutral Agree Strongly agree

1. Online support groups for eating disorders

Strongly disagree Disagree Neutral Agree Strongly agree

1. Self-help materials for eating disorders

Strongly disagree Disagree Neutral Agree Strongly agree

1. The National Eating Disorders Association (NEDA)

Strongly disagree Disagree Neutral Agree Strongly agree

1. Alliance for Eating Disorder Awareness

Strongly disagree Disagree Neutral Agree Strongly agree

1. Please list any other resources you would provide to patients with suspected or diagnosed eating disorders before discharge from the ED: ________________________________________-

Additional education and training in the following would be useful to me to apply to patients I may see in the ED:

1. Eating disorders in pediatric patients

Strongly disagree Disagree Neutral Agree Strongly agree

1. Eating disorders in adult patients

Strongly disagree Disagree Neutral Agree Strongly agree

1. Anorexia nervosa (AN-R), Restrictive Subtype

Strongly disagree Disagree Neutral Agree Strongly agree

1. Anorexia nervosa (AN-BP), Binge-Purge Subtype

Strongly disagree Disagree Neutral Agree Strongly agree

1. Severe and enduring anorexia nervosa (SE-AN)

Strongly disagree Disagree Neutral Agree Strongly agree

1. Bulimia nervosa (BN)

Strongly disagree Disagree Neutral Agree Strongly agree

1. Binge eating disorder (BED)

Strongly disagree Disagree Neutral Agree Strongly agree

1. Food addiction

Strongly disagree Disagree Neutral Agree Strongly agree

1. Other specified feeding or eating disorder (OSFED)

Strongly disagree Disagree Neutral Agree Strongly agree

1. Avoidant/restrictive food intake disorder (ARFID)

Strongly disagree Disagree Neutral Agree Strongly agree

1. Diabulimia (manipulation by diabetic patients of insulin treatments in order to lose weight)

Strongly disagree Disagree Neutral Agree Strongly agree

1. Orthorexia (obsession with eating foods that one considers healthy)

Strongly disagree Disagree Neutral Agree Strongly agree

1. Medical complications of eating disorders

Strongly disagree Disagree Neutral Agree Strongly agree

1. Diagnosis of eating disorders

Strongly disagree Disagree Neutral Agree Strongly agree

1. Assessment of patients with eating disorders in the ED

Strongly disagree Disagree Neutral Agree Strongly agree

1. Suggested criteria for hospital admission of patients with diagnoses or suspected eating disorders

Strongly disagree Disagree Neutral Agree Strongly agree

1. SCOFF Questionnaire to screen for eating disorders

Strongly disagree Disagree Neutral Agree Strongly agree

1. Resources for eating disorder patients

Strongly disagree Disagree Neutral Agree Strongly agree

1. Treatment options for patients with eating disorders post ED discharge

Strongly disagree Disagree Neutral Agree Strongly agree

1. Please indicate any other education/training that would be helpful to treating patients with suspected or diagnosed eating disorders in the ED: __________________________________________________
